# Association of serum bicarbonate with 28-day and 90-day mortality in patients with epilepsy and concurrent sepsis: a retrospective cohort study

**DOI:** 10.3389/fneur.2025.1608745

**Published:** 2025-10-17

**Authors:** Yichen Liu, Shuchang Zhou, Yingchun Mao, Qing Hu, Zhixin Li, Zhihui Li

**Affiliations:** ^1^Department of Pediatrics, The 990th Hospital of Joint Logistic Support Force of Chinese People’s Liberation Army, Zhumadian, Henan, China; ^2^Beijing Institute of Radiation and Medicine, Beijing, China

**Keywords:** bicarbonate, sepsis, epilepsy, mortality, intensive care

## Abstract

**Objectives:**

Serum bicarbonate concentration is a predictor of adverse outcomes in various diseases. However, its role in forecasting outcomes specifically for patients with epilepsy and concurrent sepsis remains unclear. This study examines the relationship between serum bicarbonate levels and 28-day and 90-day mortality in patients with epilepsy and sepsis who were admitted to the intensive care unit (ICU).

**Methods:**

Clinical data from 1,271 patients with epilepsy and concurrent sepsis were retrieved from the Medical Information Mart for Intensive Care (MIMIC)-IV database (2008–2022) for retrospective analysis. The primary outcomes measured were mortality rates at 28 and 90 days. We used multivariate Cox regression analysis, restricted cubic splines, threshold effect analysis, and survival curves to assess the impact of serum bicarbonate levels on 28-day and 90-day mortality.

**Results:**

Mortality rates for patients with epilepsy and sepsis were 21.4% at 28 days and 28.6% at 90 days. Two distinct non-linear relationships were observed between serum bicarbonate levels and mortality rates at 28 and 90 days. Below their respective threshold points, each unit increase in serum bicarbonate was associated with a decrease in mortality [hazard ratio (HR) 0.941, 95% confidence interval (CI) 0.9–0.985, *p* = 0.0084 at 28 days, and HR 0.952, 95% CI 0.915–0.99, *p* = 0.0144 at 90 days]. Above the thresholds, increases in bicarbonate levels were linked with elevated mortality risk (HR 1.1, 95% CI 0.979–1.236, *p* = 0.109 at 28 days, and HR 1.112, 95% CI 1.002–1.235, *p* = 0.0464 at 90 days). Kaplan–Meier survival analysis showed statistically significant survival differences at 28 and 90 days across serum bicarbonate levels (*p* = 0.00015), with normal levels correlating with higher survival rates.

**Conclusion:**

Two unique non-liner U-shaped relationships were identified between serum bicarbonate levels and mortality at 28 and 90 days in patients with epilepsy and concurrent sepsis. The lowest mortality rates were observed at approximately 25.0 and 25.9 mEq/L, respectively.

## 1 Introduction

Sepsis is characterized by an excessive systemic inflammatory response to infection, leading to physiological, pathological, and biochemical dysregulation that can cause multi-system and multi-organ dysfunction or failure (1). Despite significant advancements in diagnosis and treatment, sepsis remains highly prevalent, especially in high-income countries, with an incidence of approximately 270 cases per 100,000 people per year and an in-hospital mortality rate of 26% (2, 3). Globally, the incidence is estimated at 20 million cases annually, resulting in around 6 million deaths, making sepsis one of the leading causes of intensive care unit (ICU) admissions and in-hospital mortality (4, 5). The annual cost of sepsis care in the United States is estimated at $16.7 billion (6).

Sepsis is a major reason for hospital admission among patients with epilepsy (7), a common chronic neurological disorder associated with persistent risk of seizures and affecting approximately 70 million people worldwide. Epilepsy often coexists with cognitive, neuropsychiatric, and psychosocial impairments, elevating the risk of mental health conditions such as depression and anxiety, as well as increasing mortality from various diseases (8–10). Studies have reported that the relative risk of mortality in epilepsy patients is 2.87 [90% confidence interval (CI) 2.16–3.81; *I*^2^ 99.7%] and 3.66 (90% CI 2.87–4.66; *I*^2^ 97.5%), suggesting that high mortality may be associated with chronic degenerative diseases, cerebrovascular diseases, tumors, and potentially with pathophysiological mechanisms leading to sudden unexpected death in epilepsy (11). Epilepsy not only results in health complications but also imposes significant economic burdens on society and families. Epidemiological surveys estimate that the annual expenditure on epilepsy in the United States is approximately $12.5 billion (12). Multifactorial regression analysis of national hospital admissions in the United States has shown that patients with epilepsy are more likely to be transferred between hospitals [odds ratio (OR) = 1.77, 95% CI: 1.72–1.81, *p* < 0.0001], with overall costs being 4% higher (*p* < 0.0001). In light of the increased adverse outcomes of both conditions, identifying biological predictors of short-term and long-term mortality in patients with epilepsy and concurrent sepsis in the ICU is crucial for risk stratification and optimization of medical resources.

Patients with sepsis often experience hemodynamic instability, leading to inadequate tissue and organ perfusion, which in turn affects serum bicarbonate levels. Serum bicarbonate has recently been recognized as a clinical biomarker for assessing the body’s acid–base balance and predicting disease prognosis (13). Multiple studies have confirmed its role as a predictor of poor outcomes in various diseases, including all-cause mortality in type 2 diabetes (14), acute and chronic kidney diseases (15, 16), non-alcoholic liver disease (17), cardiovascular events (18), tumors (19), and ischemic cerebrovascular disease (13). However, limited research has explored the relationship between serum bicarbonate and prognosis in patients with epilepsy and concurrent sepsis. To address this gap, we conducted a retrospective cohort analysis using data from patients with epilepsy and concurrent sepsis in the ICU from the Medical Information Mart for Intensive Care (MIMIC)-IV database. This study aims to determine whether serum bicarbonate levels serve as a reliable predictor of short-term and long-term mortality in ICU patients with epilepsy and sepsis.

## 2 Methods

### 2.1 Data source

The MIMIC-IV 3.0 database^1^ is a publicly accessible database jointly established by the Computational Physiology Laboratory at the Massachusetts Institute of Technology, the Beth Israel Deaconess Medical Center (BIDMC) at Harvard Medical School, and Philips Healthcare. It includes clinical medical data from over 60,000 patients admitted to the BIDMC intensive care units between 2008 and 2022 (20). Access is granted at no cost to researchers who complete the National Institutes of Health’s web-based courses and pass the database-specific examination. As a retrospective cohort study utilizing MIMIC-IV database, without requiring patient informed consent. The authorization code (ID) for this study is 58881017.

### 2.2 Study population

**Figure 1:**
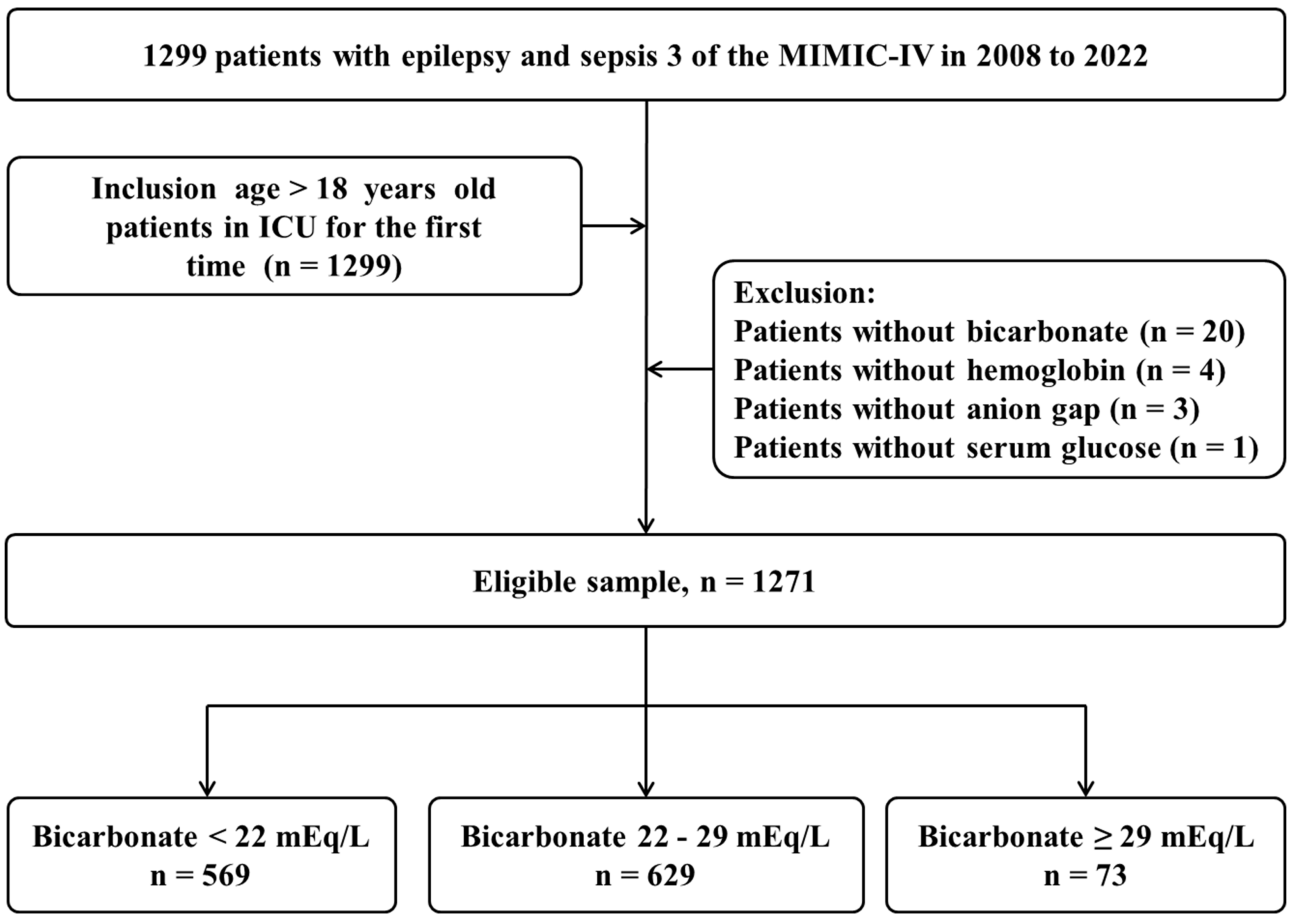
The flowchart of study participants.

The study population was identified from the MIMIC-IV 3.0 dataset using SQL (Structured Query Language). Cases were defined as epilepsy (International Classification of Diseases-9), which could appear within the first five diagnostic fields, even if not listed as the primary diagnostic. Inclusion criteria were: (1) Diagnostic consistent with the international Sepsis 3.0 diagnostic standards (21); (2) Age ≥18 years; and (3) Availability of clinical data from the patient’s first day in the ICU. Exclusion criteria were: (1) Missing serum bicarbonate data; (2) Missing anion gap results; (3) Missing data on the lowest hemoglobin concentration; and (4) Missing glucose values. After applying these criteria, clinical data from 1,271 patients were included in the analysis (Figure 1). Data on patient mortality and follow-up duration were also extracted. Missing values for pH and blood lactate levels were imputed using dummy variable methods.

### 2.3 Study variables

The baseline serum bicarbonate concentration determined was the minimum venous blood bicarbonate value measured on the first day of ICU admission for patients with epilepsy and concurrent sepsis in the MIMIC-IV database. The normal range for serum bicarbonate was considered 22–30 mEq/L (22). The primary outcomes were 28-day and 90-day mortality rates. Covariates included age, gender, race (white, black, other), lowest hemoglobin concentration, minimum platelet count, maximum white blood cell count, minimum glucose level, maximum serum chloride level, minimum sodium level, maximum blood lactate level, minimum pH from blood gas analysis, maximum anion gap, minimum blood oxygen saturation, Simplified Acute Physiology Score II (SAPS II), Oxford Acute Severity of Illness Score (OASIS), and Sequential Organ Failure Assessment (SOFA) score.

### 2.4 Statistical analysis

Continuous variables were presented as mean ± standard deviation ( $\bar{x}\pm s$ ) for normally distributed data or as median (interquartile range, IQR) for skewed data. One-way analysis of variance (ANOVA) was used for normally distributed variables, while the Kruskal–Wallis test was applied to skewed data. Categorical variables were expressed as frequency (*n*) or percentage (%) and analyzed using Chi-squared tests. The study population was divided into three groups based on serum bicarbonate levels measured on admission to ICU (<22 mEq/L, 22–29 mEq/L, ≥29 mEq/L). Additionally, restricted cubic spline (RCS) regression was conducted with knots at the 5th, 35th, 65th, and 95th percentiles of serum bicarbonate concentration to explore the relationship between bicarbonate concentration and 28-day and 90-day mortality rates. A two-piecewise Cox regression model was developed to assess the relationship between serum bicarbonate levels and mortality at 28 days and 90 days. Kaplan–Meier curves were generated to compare 28-day and 90-day mortality rates across the three groups. Both univariate and multivariate Cox regression analyses were conducted to assess the stability of the observed relationships. Adjusted and unadjusted models were used, with covariates selected for adjustment based on literature review and covariate screening analysis (23). Model I adjusted for age and gender, while Model II included additional adjustments for race, hemoglobin concentration, white blood cell count, serum chloride and sodium levels, blood lactate, pH, anion gap, SAPS II, OASIS, and SOFA scores. Results were expressed as hazard ratios (HR) with 95% CI. Threshold effect analysis was conducted to assess the relationship between admission serum bicarbonate levels and mortality at 28 days and 90 days. All statistical analyses were conducted using R software (version 4.3.1; http://www.Rproject.org), supported by R packages, and Free Statistics software (version 1.9.2; Beijing Fengrui Kelin Medical Technology Co., Ltd.) (23). A *p*-value <0.05 was considered statistically significant.

## 3 Results

### 3.1 Baseline characteristics of the study population

Table 1 summarizes the demographic and clinical characteristics as a whole, and stratified by tertiles of bicarbonate concentration of the study population. Of the 1,271 patients, 674 (53.0%) were male and 597 (47.0%) were female, with an average age of 63.0 ± 16.4 years. Within 24 h of ICU admission, the average SAPS II was 38.3 ± 13.2, OASIS averaged 34.7 ± 8.1, and the median SOFA score was 3.0 (IQR 2.0, 4.0). The mean of the lowest bicarbonate concentration measured for the whole study population on first day of ICU admission was 21.6 ± 4.8 mEq/L. The mean of the lowest bicarbonate concentration in each group were 17.6 ± 3.3, 24.2 ± 1.8, and 31.3 ± 2.9 mEq/L, respectively. Statistically significant differences were found among the groups stratified by serum bicarbonate levels for covariates such as race, lowest hemoglobin concentration, lowest platelet count, highest white blood cell count, highest serum chloride level, maximum blood lactate level, pH, maximum anion gap, SAPS II, OASIS, and SOFA (*p* < 0.05). The overall 28-day and 90-day mortality rates were 21.1 and 28.6%, respectively.

**Table 1:** Baseline characteristics of the study population.

Variables	Total (*n* = 1,271)	Serum bicarbonate (mEq/L)			*p*-value
		<22 (*n* = 569)	22–29 (*n* = 629)	≥29 (*n* = 73)	
Age, years	63.0 ± 16.4	62.3 ± 16.4	63.1 ± 16.7	66.5 ± 13.0	0.112
Gender, *n* (%)					0.707
Female	597 (47.0)	271 (47.6)	295 (46.9)	31 (42.5)	
Male	674 (53.0)	298 (52.4)	334 (53.1)	42 (57.5)	
Ethnicity, *n* (%)					0.044
White	739 (58.1)	310 (54.5)	379 (60.3)	50 (68.5)	
Black	158 (12.4)	69 (12.1)	81 (12.9)	8 (11)	
Other	374 (29.4)	190 (33.4)	169 (26.9)	15 (20.5)	
Lowest hemoglobin (g/dl)	10.2 ± 2.2	9.9 ± 2.3	10.5 ± 2.1	10.4 ± 1.9	<0.001
Lowest platelet count (×10^9^/L)	172.0 (118.0, 232.0)	160.0 (103.0, 224.0)	178.0 (127.0, 233.0)	187.0 (142.0, 272.0)	<0.001
Highest WBC count (27^9^/L)	12.6 (9.1, 17.5)	14.1(10.0, 19.7)	11.8 (8.7, 15.6)	11.2 (8.2, 15.9)	<0.001
Lowest glucose (mg/dl)	113.6 ± 39.0	114.2 ± 40.0	113.7 ± 38.8	108.4 ± 32.0	0.49
Lowest bicarbonate (mEq/L)	21.6 ± 4.8	17.6 ± 3.3	24.2 ± 1.8	31.3 ± 2.9	<0.001
Lowest sodium (mEq/L)	137.4 ± 6.1	137.0 ± 7.1	137.7 ± 5.3	137.8 ± 5.0	0.106
Highest chloride (mEq/L)	106.6 ± 7.3	108.9 ± 8.1	105.3 ± 6.0	100.8 ± 4.8	<0.001
Highest lactate-1 (mmol/L)	0.0 (0.0, 1.9)	1.0 (0.0, 2.8)	0.0 (0.0, 1.4)	0.0 (0.0, 1.4)	<0.001
Highest lactate-2 (mmol/L)	1.0 (0.0, 1.0)	0.0 (0.0, 1.0)	1.0 (0.0, 1.0)	1.0 (0.0, 1.0)	<0.001
Highest anion gap (mmol/L)	17.4 ± 5.6	19.5 ± 5.9	16.9 ± 5.5	15.4 ± 4.0	<0.001
Lowest pH-1	7.2 (0.0, 7.4)	7.2 (0.0, 7.3)	7.2 (0.0, 7.4)	7.2 (0.0, 7.4)	0.262
Lowest pH-2	0.0 (0.0, 1.0)	0.0 (0.0, 1.0)	0.0 (0.0, 1.0)	0.0 (0.0, 1.0)	0.004
Lowest SpO_2_	91.8 ± 6.2	91.6 ± 7.1	92.1 ± 5.5	90.6 ± 4.2	0.108
SAPS II	38.3 ± 13.2	41.7 ± 13.9	35.1 ± 11.7	38.5 ± 12.1	<0.001
OASIS	34.7 ± 8.1	36.1 ± 8.4	33.4 ± 7.7	35.7 ± 7.7	<0.001
SOFA	3.0 (2.0, 4.0)	3.0 (2.0, 4.0)	3.0 (2.0, 4.0)	3.0 (2.0, 4.0)	<0.001
Mortality 28 day, *n* (%)					<0.001
Survival group	1,003 (78.9)	423 (74.3)	526 (83.6)	54 (74)	
Death group	268 (21.1)	146 (25.7)	103 (16.4)	19 (26)	
Mortality 90 day, *n* (%)					<0.001
	908 (71.4)	377 (66.3)	481 (76.5)	50 (68.5)	
Death group	363 (28.6)	192 (33.7)	148 (23.5)	23 (31.5)	

Data are presented as mean ± standard deviation ( $\bar{x}\pm s$ ), median (IQR), and *N* (%). WBC, white blood cell; pH, potential Hydrogen; SpO_2_, oxygen saturation; OASIS, Oxford Acute Severity of Illness Score; SAPS II, Simplified Acute Physiology Score II; SOFA, Sequential Organ Failure Assessment.

### 3.2 Relationship between serum bicarbonate levels and survival outcomes

Univariate Cox regression analysis identified risk factors significantly associated with 28-day and 90-day mortality, including age, lowest hemoglobin concentration, highest white blood cell count, minimum glucose level, blood lactate, anion gap, SAPS II, OASIS, SOFA, and serum bicarbonate concentration (*p* < 0.05). However, gender, race, and other test indices were not associated with 28-day and 90-day mortality rates (Table 2).

**Table 2:** Relationship between single-variable factors and 28-day and 90-day mortality rates in patients with epilepsy and concurrent sepsis.

Variables	Mor-28 day HR (95%CI)	Mor-28 day *p*-value	Mor-90 day HR (95%CI)	Mor-90 day *p*-value
Age, years	1.03 (1.02, 1.04)	<0.001	1.03 (1.02, 1.04)	<0.001
Gender				
Male vs. female	0.87 (0.68, 1.1)	0.251	0.94 (0.76, 1.15)	0.551
Ethnicity				
White	Ref	0.416	Ref	0.524
Black	0.98 (0.67, 1.44)	0.932	1.04 (0.76, 1.44)	0.791
Other	1.19 (0.91, 1.55)	0.204	1.14 (0.91, 1.44)	0.253
Lowest hemoglobin (g/dl)	0.93 (0.88, 0.99)	0.013	0.92 (0.88, 0.96)	<0.001
Lowest platelet count (×10^9^/L)	0.9989 (0.9977, 1.0002)	0.106	0.9987 (0.9976, 0.9998)	0.025
Highest WBC count (×10^9^/L)	1.02 (1.01, 1.03)	<0.001	1.02 (1.01, 1.03)	<0.001
Lowest glucose (mg/dl)	1.002 (1.0009, 1.003)	<0.001	1.0039 (1.0015, 1.0063)	0.002
Lowest sodium (mEq/L)	0.9973 (0.9776, 1.0175)	0.793	0.9971 (0.98, 1.0146)	0.747
Highest chloride (mEq/L)	0.9985 (0.9818, 1.0155)	0.859	1.0017 (0.9873, 1.0164)	0.815
Lowest bicarbonate (mEq/L)	0.93 (0.91, 0.95)	<0.001	0.94 (0.92, 0.96)	<0.001
Highest lactate-1 (mmol/L)	1.19 (1.15, 1.23)	<0.001	1.16 (1.12, 1.2)	<0.001
Highest lactate-2 (mmol/L)	0.71 (0.56, 0.91)	0.006	0.86 (0.7, 1.06)	0.147
Highest anion gap (mmol/L)	1.06 (1.04, 1.08)	<0.001	1.05 (1.03, 1.06)	<0.001
Lowest pH-1	0.02 (0.01, 0.08)	<0.001	1.02 (0.99, 1.05)	0.15
Lowest pH-2	0.71 (0.55, 0.91)	0.007	0.85 (0.69, 1.05)	0.125
Lowest SpO_2_	0.96 (0.95, 0.98)	<0.001	0.97 (0.96, 0.98)	<0.001
SAPS II	1.05 (1.04, 1.06)	<0.001	1.05 (1.04, 1.05)	<0.001
OASIS	1.07 (1.05, 1.08)	<0.001	1.06 (1.05, 1.08)	<0.001
SOFA	1.18 (1.11, 1.24)	0.002	1.17 (1.12, 1.23)	<0.001
Tertile of lowest bicarbonate (mEq/L)				
<22	1.69 (1.32, 2.18)	<0.001	1.57 (1.27, 1.95)	<0.001
22–29	Ref	<0.001	Ref	<0.001
≥29	1.6 (0.98, 2.61)	0.006	1.38 (0.89, 2.15)	0.147

WBC, white blood cell; pH, potential Hydrogen; SpO_2_, oxygen saturation; OASIS, Oxford Acute Severity of Illness Score; SAPS II, Simplified Acute Physiology Score II; SOFA, Sequential Organ Failure Assessment; HR, hazard ratio; CI, confidence interval. *p* value calculated using Wald’s test.

Multivariate Cox regression analysis indicated that serum bicarbonate, as a continuous variable, was associated with reduced the 28-day and 90-day mortality rates in all models (unadjusted model, Model 1, and the fully adjusted Model 2) (Table 3). However, in Model 2, the associations were not statistically significant.

**Table 3:** Relationship between serum bicarbonate levels (as a continuous variable and categorized) and 28-day and 90-day mortality rates using adjusted and non-adjusted models.

Serum bicarbonate	Non-adjusted model		Model 1		Model 2	
	HR (95% CI)	*p*value	HR (95% CI)	*p*value	HR (95% CI)	*p*value
28-day mortality						
Bicarbonate (mEq/L)	0.93 (0.91–0.95)	<0.001	0.93 (0.9–0.95)	<0.001	0.98 (0.94–1.02)	0.254
<22 mEq/L	1.69 (1.32–2.18)	<0.001	1.75 (1.36–2.25)	<0.001	1.16 (0.85–1.58)	0.34
22–29 mEq/L	1 (Ref)		1 (Ref)		1 (Ref)	
≥29 mEq/L	1.6 (0.98–2.61)	0.06	1.49 (0.91–2.43)	0.112	1.54 (0.92–2.59)	0.089
Trend	0.73 (0.6–0.91)	0.004	0.71 (0.57–0.87)	0.001	1 (0.77–1.3)	0.994
90-day mortality						
Bicarbonate (mEq/L)	0.94 (0.92–0.96)	<0.001	0.93 (0.91–0.95)	<0.001	0.98 (0.95–1.01)	0.213
<22 mEq/L	1.57 (1.27–1.95)	<0.001	1.62 (1.31–2.01)	<0.001	1.1 (0.85–1.43)	0.463
22–29 mEq/L	1 (Ref)		1 (Ref)		1 (Ref)	
≥29 mEq/L	1.38 (0.89–2.15)	0.147	1.28 (0.83–1.99)	0.265	1.34 (0.85–2.12)	0.212
Trend	0.75 (0.63–0.9)	0.002	0.72 (0.6–0.86)	<0.001	1 (0.8–1.24)	0.992

Model 1: adjusted for age and gender; Model 2: adjusted for age, gender, ethnicity, hemoglobin, sodium, WBC count, chloride, lactate, anion gap, pH, SAPS II, OASIS, SOFA. WBC, white blood cell; pH, potential Hydrogen; SpO_2_, oxygen saturation; OASIS, Oxford Acute Severity of Illness Score; SAPS II, Simplified Acute Physiology Score II; SOFA, Sequential Organ Failure Assessment; HR, hazard ratio; CI, confidence interval. *p* value calculated using Wald’s test.

When serum bicarbonate was categorized, patients with levels <22 mEq/L showed significantly higher 28-day and 90-day mortality rates compared to the baseline group (22–29 mEq/L) (Table 3). In the unadjusted model, the mortality risk increased by 69% for 28-day mortality (HR = 1.69, 95% CI 1.32–2.18, *p* < 0.001) and 57% for 90-day mortality (HR = 1.57, 95% CI 1.27–1.95, *p* < 0.001). Model 1 (adjusted for age and gender) showed similar increases of 75% (HR = 1.75, 95% CI 1.36–2.25, *p* < 0.001) and 62% (HR = 1.62, 95% CI 1.31–2.01, *p* < 0.001), respectively. In Model 2 (adjusted for all potential confounders), the risk increase remained, but was not statistically significant. Patients with serum bicarbonate levels ≥29 mEq/L showed higher 28-day and 90-day mortality rates across all models, but these results were not statistically significant (Table 3).

### 3.3 Non-linear association between serum bicarbonate and 28-day and 90-day mortality rates

**Figure 2:**
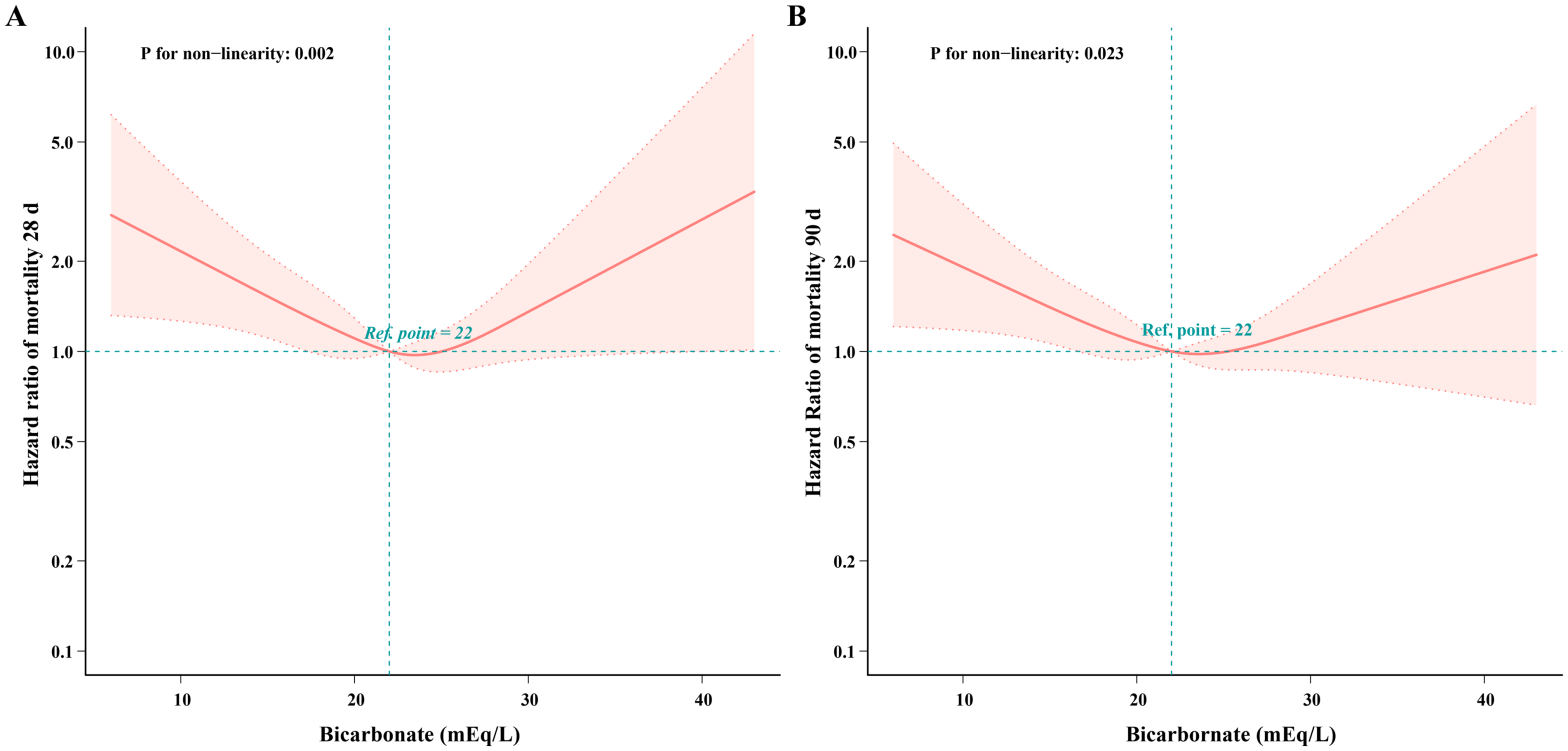
Non-linear relationship between serum bicarbonate concentrations and 28-day and 90-day mortality rates in patients with epilepsy and concurrent sepsis. **A** 28 days; **B** 90 days. Bicarbonate scaled up to 100%.

Multivariable-adjusted restricted cubic splines analysis suggested a U-shaped association between levels of serum bicarbonate on admission and risk of 28-day and 90-day mortality rates (*p* for nonlinearity <0.05, Figure 2). Specifically, this analysis indicated that as admission serum bicarbonate levels change, the associated mortality rates do not follow a straightforward linear pattern; instead, they exhibit a complex, curvilinear relationship.

The piecewise multivariable Cox regression model with two different slopes showed a significant likelihood ratio of less than 0.05, and identified a threshold point at approximately 25.0 mEq/L for 28 days and 25.9 mEq/L for 90 days mortality. This indicates that the relationship is non-linear, with two separate distinct hazard ratios both above and below the turning point. Below the respective threshold, rising bicarbonate levels are correlated with a significantly reduced risk of mortality at both 28 days (HR = 0.941, *p* < 0.05) and 90 days (HR = 0.952, *p* < 0.05). Above the threshold, rising bicarbonate levels are linked to a non-significant rise in 28-day mortality risk (HR = 1.1, 95% CI 0.979–1.236, *p* > 0.05), yet a statistically significant 11.2% increase in 90-day mortality risk (HR = 1.112, 95% CI 1.002–1.235, *p* < 0.05) (Table 4).

**Table 4:** Threshold effect analysis of serum bicarbonate levels by different groups.

Category	HR	95% CI	*p*value
28-day mortality			
Turning point (mEq/L)	25.014	24.653, 25.374	
Bicarbonate <25.014 mEq/L	0.941	0.9, 0.985	0.0084
Bicarbonate >25.014 mEq/L	1.1	0.979, 1.236	0.109
Likelihood ratio test			0.008
90-day mortality			
Turning point (mEq/L)	25.883	25.493, 26.272	
Bicarbonate <25.883 mEq/L	0.952	0.915, 0.990	0.0144
Bicarbonate >25.883 mEq/L	1.112	1.002, 1.235	0.0464
Likelihood ratio test			0.021

Data presented are HRs and 95% CI.

Adjusted for age, gender, ethnicity, hemoglobin, sodium, WBC count, chloride, lactate, anion gap, pH, SAPS II, OASIS, SOFA. WBC, white blood cell; pH, potential Hydrogen; SpO_2_, oxygen saturation; OASIS, Oxford Acute Severity of Illness Score; SAPS II, Simplified Acute Physiology Score II; SOFA, Sequential Organ Failure Assessment; HR, hazard ratio; CI, confidence interval.

### 3.4 Kaplan–Meier survival curve analysis

**Figure 3:**
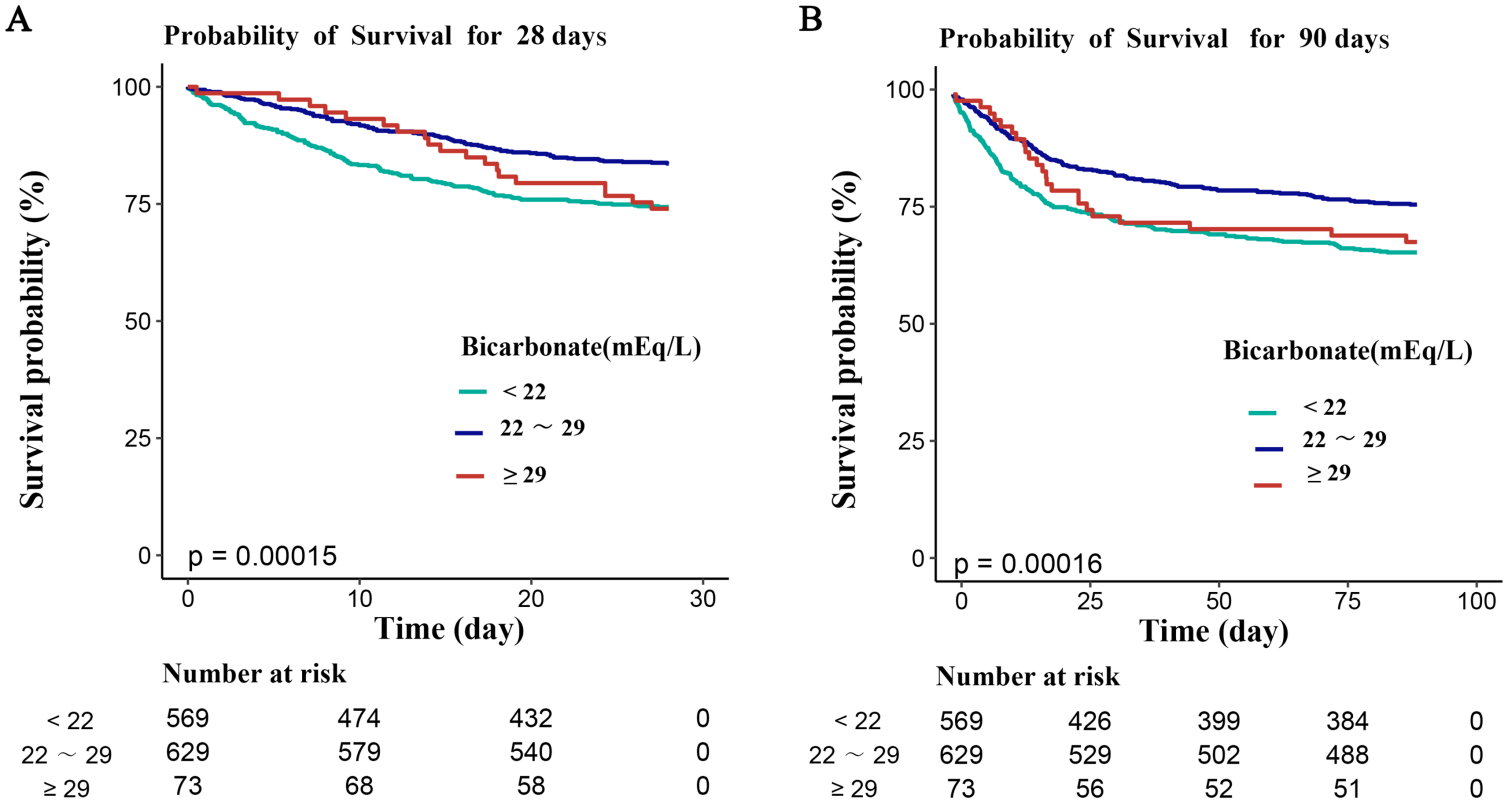
Survival curves showing the relationship between serum bicarbonate levels measured on ICU admission and 28-day and 90-day mortality rates. **A** 28 days; **B** 90 days.

Kaplan–Meier survival curves, plotted using admission serum bicarbonate levels as a categorical variable, showed significant differences in both 28- and 90-day survival rates between groups (*p* < 0.001 and *p* < 0.001, respectively; Figure 3). Compared with the baseline serum bicarbonate group (22–29 mEq/L), patients with serum bicarbonate concentrations below 22 mEq/L or above 29 mEq/L demonstrated a higher risk of mortality rates, highlighting the association between serum bicarbonate homeostasis and survival (Figure 3).

## 4 Discussion

This retrospective cohort study demonstrated that serum bicarbonate levels measured at time of ICU admission are an independent prognostic factor for 28-day and 90-day mortality rates in patients with epilepsy and concurrent sepsis. Results from the multivariable Cox regression models, Kaplan Meier survival curves, cubic spline analysis, and threshold effect analysis demonstrate a non-linear U-shaped relationship between bicarbonate concentration and mortality. Serum bicarbonate analysed as a continuous variable showed that lower serum bicarbonate concentration significantly increased 28-day and 90-day mortality rates. The finding of reduced short- and long-term mortality with increasing bicarbonate levels, when treated as a continuous variable, was statistically non-significant and likely influenced by the relatively small sample size of patients with elevated serum bicarbonate concentrations. When serum bicarbonate was categorized, patients with levels <22 mEq/L demonstrated significantly higher risks of both short-term and long-term mortality compared to those in the 22–29 mEq/L range. Similarly, levels ≥29 mEq/L were associated with increased mortality risk, though these results were not statistically significant. Threshold effect analysis identified a turning point 25.0 mEq/L for 28-day and 25.9 mEq/L for 90-day mortality, where increasing bicarbonate levels below this point are associated with reduced mortality, and increasing levels above this point are linked to increased mortality. These results align with previous findings regarding the U-shaped relationship between bicarbonate and mortality among critically ill patients. Libório et al. (24), reported a U-shaped relationship between serum bicarbonate levels and in-hospital mortality among critically ill patients. Specifically, their study identified two serum bicarbonate concentration nodes, at 25 mEq/L and 30 mEq/L, that correspond to this U-shaped pattern. These observations are consistent with the findings of the current studies. Notably, no previous studies have explored the specific relationship between serum bicarbonate levels and 28-day or 90-day mortality rates in patients with epilepsy and concurrent sepsis.

The international Sepsis-3 consensus defines sepsis as “life-threatening organ dysfunction caused by a dysregulated host response to infection” (21). This dysfunction leads to hemodynamic failure and abnormal cell metabolism, leading to anaerobic glycolysis in terminal organs and systemic lactic acidosis (25). Sepsis-related organ dysfunction often involves liver and kidney impairment, which exacerbates systemic acidosis (26). Liver damage reduces lactate clearance, while acute kidney injury leads to hydrogen ion retention and bicarbonate loss, intensifying acid–base imbalance (25). Metabolic acidosis, characterized by reduced serum bicarbonate levels, disrupts acid–base homeostasis and contributes to adverse clinical outcomes. Lower serum bicarbonate concentrations have been linked to a higher incidence of acute cardiovascular events and mortality rate within 28 days due to ischemic cardiogenic shock (25, 27). They have also been associated with a higher risk of progression to kidney transplantation or a 50% reduction in glomerular filtration rate in children with acute kidney disease (28). Additionally, they have been significantly correlated with all-cause and cancer mortality rates in patients with type 2 diabetes (19). Low bicarbonate may be related to acidosis, which increases intracellular acidity and releases intracellular protein-bound iron, leading to the formation of hydroxyl radicals that cause oxidative stress and tissue damage (29). Moreover, acidosis activates inflammatory gene expressions in endothelial cells, mediating endothelial plasma leakage, leukocyte recruitment, and exacerbated tissue injury (30). Given these mechanisms, serum bicarbonate is an effective clinical predictor of mortality in sepsis patients, with lower levels strongly associated with increased in-hospital mortality rates compared to those with normal or higher (31). Conversely, elevated serum bicarbonate levels (above 30 mEq/L) have also been associated with increased mortality risks by 21% in critically ill patients (24), Bicarbonate depletion reflects worsening sepsis-related organ dysfunction related to sepsis, whereas bicarbonate excess may suggest metabolic alkalosis, resulting in an increase in pH, a leftward shift of the oxygenated hemoglobin dissociation curve, electrolyte imbalance, and supraventricular or ventricular arrhythmias (32). It thus serves as an effective clinical predictor of mortality in sepsis patients.

Additionally, bicarbonate consumption reflects lactic acid accumulation during seizures, leading to metabolic acidosis, simultaneously activating acid-sensing ion channels (ASIC1a) in both neurons and astrocytes, thereby increasing the frequency of spontaneous seizures. Meanwhile, ASIC1a promotes the activation of the N-methyl-D-aspartate receptor (NMDAR) and exacerbates neuronal death caused by acidosis, leading to calcium ion influx (33, 34). Olaciregui Dague et al. (35), reported significantly lower serum bicarbonate levels (median: 22.6 mmol/L, range 7.1–33.2 mmol/L) in 165 patients with epilepsy following seizures compared with 58 controls with non-epileptic seizure-like events. In the other hand, excessive serum bicarbonate levels can lead to metabolic alkalosis and may strongly predict mortality and adverse functional outcomes in patients with status epilepticus (36). Previous studies have reported that HCO3− plays an important role in neuronal plasticity, as the transmembrane gradient of HCO3− and Cl− determines whether the γ-aminobutyric acid (GABA) ergic signal is depolarizing and excitatory or hyperpolarizing and inhibitory (37, 38). During an epileptic seizure, the consistent depolarization driven by the GABAAR-mediated HCO3− current boosts the activity-dependent Cl− influx into neurons, thereby directly triggering excitation in this state (39). In this study, Kaplan–Meier survival curve analyses revealed that serum bicarbonate levels below 22 mEq/L or above 29 mEq/L were associated with increased mortality at both 28 days and 90 days, corroborating earlier findings. The research demonstrated not only that both depleted and excessive bicarbonate levels are associated with mortality in both sepsis and epilepsy but also reinforced the utility of serum bicarbonate as a biomarker in patients with sepsis and concurrent epilepsy. Individuals with serum bicarbonate levels below 22 mEq/L or above 29 mEq/L can be identified as high-risk at an early stage. This early identification allows for more targeted and timely interventions, such as closer monitoring of vital signs, more frequent laboratory tests, and early adjustment of treatment plans. However, given the complexity of the pathogenesis of epilepsy and concurrent sepsis, a single biomarker may not be adequate for accurately predicting prognosis. Utilizing a combination of serum bicarbonate alongside markers such as procalcitonin, C-reactive protein, and lactate could enhance the precision of risk assessment and prognosis prediction.

This study possesses several advantages. Firstly, it employs data from the well-established MIMIC-IV 3.0 database, which is a rich and reliable source of ICU patient information. Moreover, this research employed appropriate statistical methods to verify the curve relationships of the association among serum bicarbonate concentration and short-term or long-term mortality rates, rather than a simple straight-line relationship. Lastly, we have identified the threshold for the smooth curve, which may contribute to suggesting to ICU physicians a better understanding and management of changes in the serum bicarbonate threshold of critically ill patients. Nevertheless, some limitations need to be taken into account. Firstly, as with other retrospective cohort studies, residual confounding may persist despite comprehensive adjustments for known confounding factors. Secondly, the data were solely sourced from the single-center MIMIC-IV 3.0 database, limiting the generalizability of the findings to broader populations. Thirdly, only the lowest serum bicarbonate levels during the first day of admission to ICU were analyzed. This approach does not account for dynamic changes in serum bicarbonate levels throughout the ICU stay, which may have significant implications for patient outcomes. Specifically, fluctuations in bicarbonate levels over time might reflect different pathophysiological processes effects that were not captured in our analysis. Future studies should consider longitudinal measurements of serum bicarbonate levels to better understand their prognostic significance and potential role in guiding clinical interventions.

## 5 Conclusion

There were two separate non-linear U-shaped relationships between serum bicarbonate levels at ICU admission and both short-term and long-term mortality rates in patients with concurrent sepsis and epilepsy rates. Compared to the normal range of serum bicarbonate concentration, both the 28-day and 90-day mortality risks were increased when the serum bicarbonate level deviates from the normal value. The optimal serum bicarbonate values associated with the lowest short-term and long-term mortality risks are 25.0 and 25.9 mEq/L, respectively. These results suggest a useful marker for risk stratification of patients with epilepsy and concurrent sepsis in the ICU, and underscore the importance of maintaining optimal serum bicarbonate levels to improve survival outcomes in these patients.

## Data Availability

The original contributions presented in the study are included in the article/supplementary material, further inquiries can be directed to the corresponding author.

## Footnotes

^1^ https://physionet.org/content/mimiciv/3.0/
